# Role of nutrient concentrations and water movement on diatom’s productivity in culture

**DOI:** 10.1038/s41598-018-37611-6

**Published:** 2019-02-06

**Authors:** Ida Orefice, Margherita Musella, Arianna Smerilli, Clementina Sansone, Raghu Chandrasekaran, Federico Corato, Christophe Brunet

**Affiliations:** 1 Stazione Zoologica Anton Dohrn, Istituto Nazionale di Biologia, Ecologia e Biotecnologie Marine, Villa comunale, 80121 Napoli, Italy; 2 Present Address: CAS in Marine Biology, Faculty of Marine Sciences, Annamalai University, Parangipettai, 608502 Tamil Nadu India

## Abstract

Microalgal growth maximization is becoming a duty for enhancing the biotechnological fate of these photosynthetic microorganisms. This study, based on an extensive set of data, aims to revisit diatom’s cultivation in laboratory with the objective to increase growth rate and biomass production. We investigated the growth ability and resource requirements of the coastal diatom *Skeletonema marinoi* Sarno & Zingone grown in laboratory in the conventional f/2 medium with aeration and in two modified conditions: (i) the same medium with water movement inside and (ii) an enriched medium with the same water movement. Results revealed that, by doubling the concentration of phosphate, silicate, microelements and vitamins, growth rate was successfully enhanced, preventing phosphate or silicate limitation in the f/2 culture medium. Yet, irrespective of the media (f/2 or enriched one), water movement induced an increase of growth efficiency compared to aeration, affecting nutrients’ requirement and consumption by diatoms. This study is an important step for enhancing diatom biomass production, reducing its cost, as required in the blue biotechnology context.

## Introduction

The interest in maximizing microalgal production is nowadays growing since this group of photosynthetic microbes is greatly promising as natural sources of many products/processes useful for various biotechnological applications regarding the environment, energy, health, food, or cosmetics^1^. Numerous researchers are facing this issue, applying different approaches, from genetic^2^ to environmental manipulation, such as light modulation^3,4^. Together with light and temperature, nutrients are one of the most important key drivers of phytoplankton growth^5,6^. Microalgae require macro-, micronutrients and vitamins for growth. Macronutrients correspond to nitrogen (N) and phosphorus (P), while micronutrients correspond to trace metals (e.g. iron, manganese, cobalt, etc.). In addition, vitamins such as thiamine, biotin and cobalamin (vitamins B_1_, B_7_ and B_12_, respectively) are also needed since some microalgal species are not able to synthesize them^7^. Diatoms require silicon (Si), which is involved in building the outer cell wall, or frustule^8^. Yet, silicate metabolism is tightly coupled with cell cycle^9^ and appears linked to respiration^10,11^.

Nitrogen is required for biosynthesis of many molecules such as amino acids, nucleic acids, lipids and some sugars; the bulk of assimilated N being used for proteins and nucleic acids^12^.

Phosphorus is a component of the backbone of DNA and RNA, with the elevated presence of sugar phosphate. RNA accounts for more than 50% of the total P-content of phytoplankton cells^13^, while RNA pool is extremely variable in cells^14^. Phosphorus is also present in phospholipids and ATP^13^ while cells can store P under the form of polyphosphate^13,15^.

The molar stoichiometry of these elements in phytoplankton is known as “Redfield ratio”^16^, even though the relative contribution of these elements in cells varies with the demand for each of these components. Cell’s requirement for these major elements depends on microalgal diversity^15,17^, as well as on many environmental factors, such as light or temperature^5,12,13,18–21^. Uptake and requirements of nutrients directly affect growth process, as reported in the Growth rate hypothesis (GRH)^13,14,19,22^. The latter states “differences in organismal C/N/P ratios are caused by differential allocations to RNA necessary to meet the protein synthesis demands of rapid rates of biomass growth and development”^14^.

Another crucial aspect for microalgal cultivation is the shear stress^23^ and small-scale turbulence^24–26^. Mixing circulate fluid is essential for microalgal cultivation helping to maintain homogeneous suspension and ensures that microalgal cells have access to the resources, such as light or nutrients. Aeration is usually applied to microalgal culture supplying CO_2_, removing O_2_ produced by photosynthesis and helping in fluid circulation. While air bubbling can regulate pH variations during cultivation^27,28^, it is also known to be detrimental to microalgal cells^29^.

Starting with the observations of growth limitation by nutrient (P and/or Si) of the coastal diatom *Skeletonema marinoi* Sarno & Zingone, our study aimed to enhance growth rate of this nutritionally-rich species^3,30^, relevant for aquaculture purposes^31–34^. The objectives of this study are (i) to investigate the modulation of growth rate and (ii) to address the GRH quantifying the macronutrient uptake of this species grown in the conventional f/2 medium and in an enriched medium. The third objective is to enhance growth of this species addressing the question of mixing, comparing aeration with water movement created by an aquarium wave-maker pump.

Growth rate and uptake of nitrate, phosphate, and silicate has been estimated during 21-laboratory microcosm experiments carried out on this diatom. Cellular RNA content enriched the dataset and allowed to investigate the link between growth rate, nutrient uptake and physiological state of the cells.

## Materials and Methods

### The model species *Skeletonema marinoi*

The species *S*. *marinoi* is very abundant during the spring bloom in temperate waters when its concentration can reach millions of cells per litre in the photic zone. *Skeletonema marinoi* (CCMP 2092), a cosmopolitan centric diatom, was used as a model species since its high growth capacity, its biotechnology interesting biochemical profile^3,30^ and relevant role for aquaculture applications^31–34^ and the information already available on the biology of this species^3,4,35^,

### Experimental data set

Our study is based on 291 data, obtained from 21 experiments conducted on the coastal centric diatom *S*. *marinoi* (Table 1). Experiments were conducted on axenic cultures. All experiments were carried out at 20 °C with autoclaved seawater, pre-filtered through a 0.7 µm GF/F glass-fiber filter (Whatman™, Whatman International Ltd, Maidstone, UK). Light was provided by a custom-built LED illumination system (European patent registration number: EP13196793.7), allowing to modulate the spectral composition and light intensity^36^. Light intensity was measured inside each flask by using a laboratory PAR 4π sensor (QSL 2101, Biospherical Instruments Inc., San Diego, CA, USA).

**Table 1:** Experimental data set.

Medium	Water movement/aeration	Growth phase	Experimental strategy	Light conditions
f/2	Aeration (air-bubbling)	Exponential (n = 28) Stationary (n = 6) Death (n = 7)	Pre-acclimated	Blue (88, 150, 250, 450) Blue + red (150) Sinusoidal/Square Photoperiod: 12 h:12 h
f/2	Water movement (wave maker pump)	Exponential (n = 15) Stationary (n = 1) Death (n = 6)	Pre-acclimated	White (150) Sinusoidal Photoperiod: 12 h:12 h
Enriched medium	Water movement (wave maker pump)	Exponential (n = 101) Stationary (n = 12) Death (n = 42)	Pre-acclimated (n = 122) Shift (n = 33)	White (10, 88, 150, 300, 600) blue (150); red (150); green (150) Photoperiod: 12 h:12 h; 24 h:0 h; 0 h:24 h Sinusoidal/Square

Light intensity in brackets is in µmol m^−2^ s^−1^. Sinusoidal is for sinusoidal light distribution, square is for wave square light distribution. Blue, red, green are for monochromatic blue, red, green wavelengths, respectively; blue + red is for blue light condition over imposed with red light peaks. White is for white light composed by blue, red and green (50, 5, 45%, respectively). n is the number of data for each class.

The first set of data corresponded to *S*. *marinoi* cultivated in the classical f/2 medium for diatom’ s cultivation^37^ with an addition of silicate. The second experimental data set originated from *S*. *marinoi* cultivated in an enriched medium, characterized by twice the concentrations of phosphate, silicate, metals, and vitamins compared to the conventional f/2 medium.

Two conditions of physical motion in the fluid were compared: aeration (air bubbling) *vs*. water movement using an aquarium wave maker pump (Sunsun, JVP-110) (Table 1).

The dataset was discriminated respect to the growth phases: exponential phase, stationary phase or death (Table 1), and the different light conditions in term of light intensity, spectral composition and distribution over time were taken into account. Moreover, we separated the experiments carried out on pre-acclimated cells to the experimental light condition *vs*. shifted cells from a pre-acclimation light to the experimental light conditions (Table 1).

### Nutrient analysis and nutrient uptake rate estimation

Samples for determining nutrient concentrations were collected in 20 mL polyethylene vials, and quickly frozen and stored at −20 °C. Ammonium, nitrate, nitrite, silicon and phosphate concentrations were determined using a Technicon Auto Analyzer following classical methods^38^.

Nutrient concentrations sampled every day were therefore used to estimate the daily uptake of nutrients by cells:

1 $${\rm{Nu}}=\frac{{{\rm{N}}}_{{\rm{n}}}-{{\rm{N}}}_{{\rm{n}}-1}}{{{\rm{C}}}_{{\rm{n}}}-{C}_{{\rm{n}}-1}}$$

where Nu is the nutrient uptake (pmol cell^−1^ day^−1^), N_n_ the nutrient concentration at day n and C_n_ the cell concentration at day n.

### Cell concentration and growth rate

To assess cell density, 2 mL of cell suspension were collected from each flask and fixed with Lugol’s iodine solution (1.5% v/v). One mL of this solution was used to fill a Sedgewick Rafter counting cell chamber. Cells were then counted using a Zeiss Axioskop 2 Plus light microscope (Carl Zeiss, Göttingen, Germany).

The growth rate was estimated from cell concentration measurements using the following equation:

2 $$\mu ({{\rm{d}}}^{-1})=\frac{{\rm{l}}{\rm{n}}({{\rm{C}}}_{{\rm{n}}-1}/{{\rm{C}}}_{{\rm{n}}})}{{{\rm{t}}}_{{\rm{n}}}-{t}_{{\rm{n}}-1}}$$

where µ is the growth rate, C_n_ and C_n−1_ are cell concentrations (mL^−1^) at day n − 1 (t_n−1_) and day n (t_n_).

Assuming that every individual cell doubles with every cell cycle, we estimated the proliferation of cells by the equation:

3 $${{\rm{C}}}_{{\rm{n}}}={{\rm{C}}}_{{\rm{n}}-1}{2}^{{\rm{tf}}}$$

where C_n_ is the cell concentration at day n, C_n−1_ is the cell concentration at day n − 1, and f is the frequency of cell cycles per unit of time.

### Intracellular phosphorus partition estimation

The P uptake rate per cell (see above, pmol cell^−1^ day^−1^) was transformed in content of P per cells (pg cell^−1^), using the phosphorus molar weight, assuming that all acquired phosphorus is internally used by cells. With the aim to discriminate the phosphorus allocation in cells, we proceeded in the following way: we evaluated the P-containing RNA content applying the data P = 0.091 g. dry g RNA^−1^ ^12^ to the cellular RNA concentration (see below). Then, we obtained the no-RNA-P content per cells (removing the P-containing RNA from the total P content per cell estimated previously). Two other pools of cellular P can be discriminated^12^: the total inorganic phosphorus and the other P-containing compounds (i.e., excluding RNA and inorganic P pools). From the data reported in Geider and La Roche^12^, we applied a value of 20% on the total no-RNA-P content to obtain the total inorganic phosphorus in cells while the other 80% accounts for the other P-containing compounds^12^.

### RNA analysis

Fifty mL of each of the triplicates were centrifuged at 4000 rpm (3399 g) for 20 min at 4 °C (DR15P centrifuge, B. Braun Biotech International, Melsungen, Germany). The supernatant was discarded and the pellet was transferred to a 2 mL Eppendorf tube and centrifuged at 14000 rpm (21952 g) for 15 min at 4 °C (5417 R centrifuge, Eppendorf, Hamburg, Germany). The pellet was re-suspended in 800 μL of TRIzol (Invitrogen, Carlsbad, CA, USA), incubated for 2–3 min at 60 °C until it was completely dissolved. Samples were frozen in liquid nitrogen and kept at −80 °C until analysis. The total RNA has been extracted following the already described procedure^39^. DNase treatment was carried out using DNase I recombinant, RNase-free (Roche, Basel, Switzerland). Then, total RNA sample was purified and concentrated using RNeasy MinElute Cleanup Kit (Qiagen, Venlo, Netherlands) and eluted in 20 µL RNase-free water. Concentration of resulting RNA was evaluated by absorbance at 260 nm (ND-1000 Spectrophotometer; NanoDrop Technologies, Wilmington, DE, USA) and RNA integrity was checked by agarose gel electrophoresis.

### Statistical analysis

Statistical analysis was performed using Past 3^40^. We performed calculations of mean, standard deviation, variance, the coefficient of variation (CV). Mann-Whitney U test was applied to compare the data between different discriminated groups. Spearman correlation has been applied to analyse the trend between our variables separating or including different experiments.

## Results and Discussion

Although it is known that light significantly affects growth capacity and thus nutrient requirement^6,11,12^, our study did not depicted large effects of light on nutrient uptake. Nitrate (NO_3_^−^) and silicate (SiO_4_^4−^) uptake did not significantly vary under the different light conditions, while under prolonged darkness NO_3_^−^ uptake decreased significantly (p < 0.001). On the opposite, phosphate (PO_4_^3−^) uptake increased under low light compared to moderate and high light (p < 0.01). Since the very circumscribed effects of light and, in order to prevent a bias in the further analysis, we excluded the data conditioned by light effect from the dataset.

### Growth and nutrient uptake of *S*. *marinoi* grown in f/2 medium with aeration

**Figure 1:**
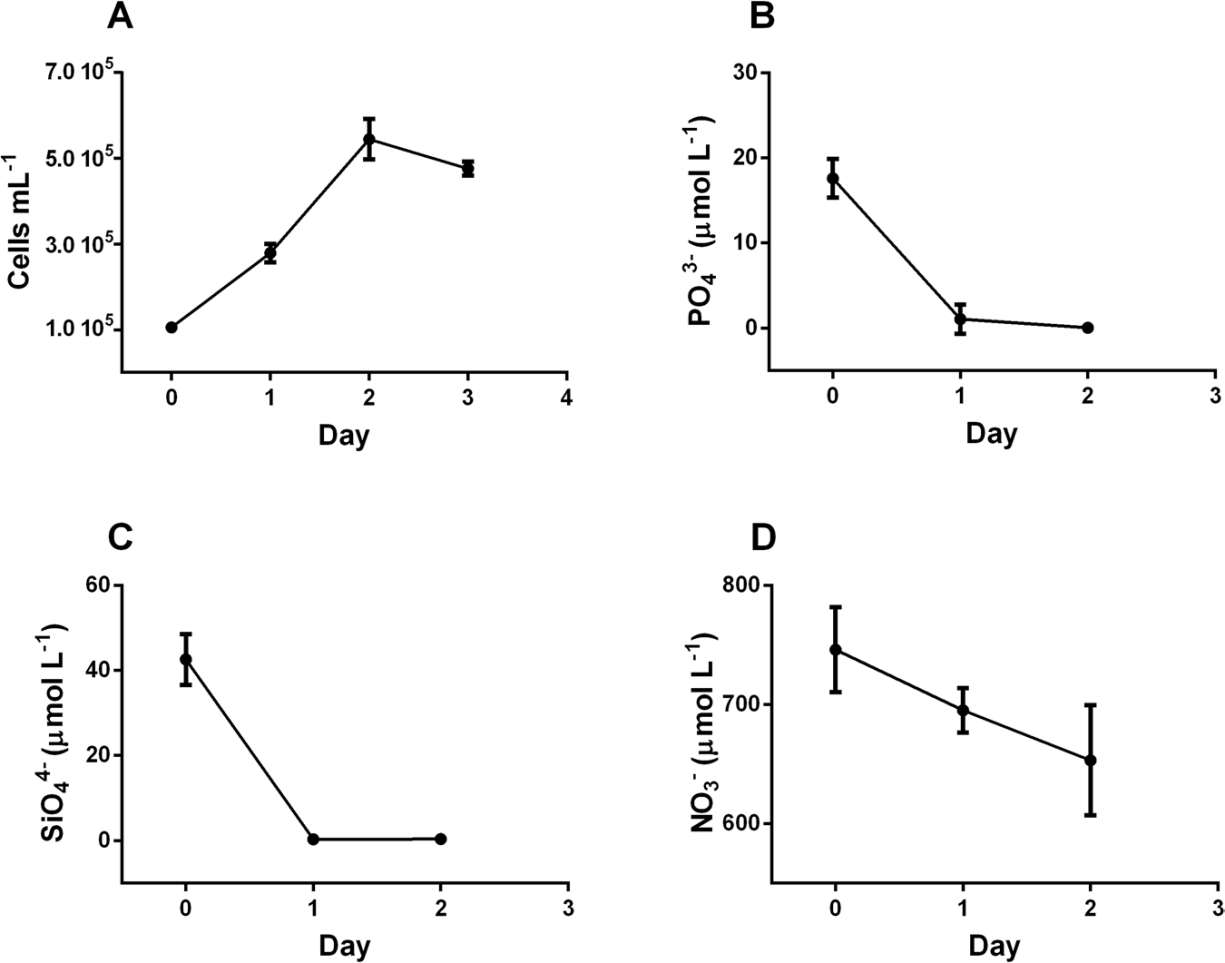
Cell concentration (**A**), cells mL^−1^) and PO_4_^3−^ (**B**), SiO_4_^4−^ (**C**), and NO_3_^−^ (**D**) concentrations (µmol L^−1^) along the exponential growth phase in the original f/2 medium with aeration.

**Figure 2:**
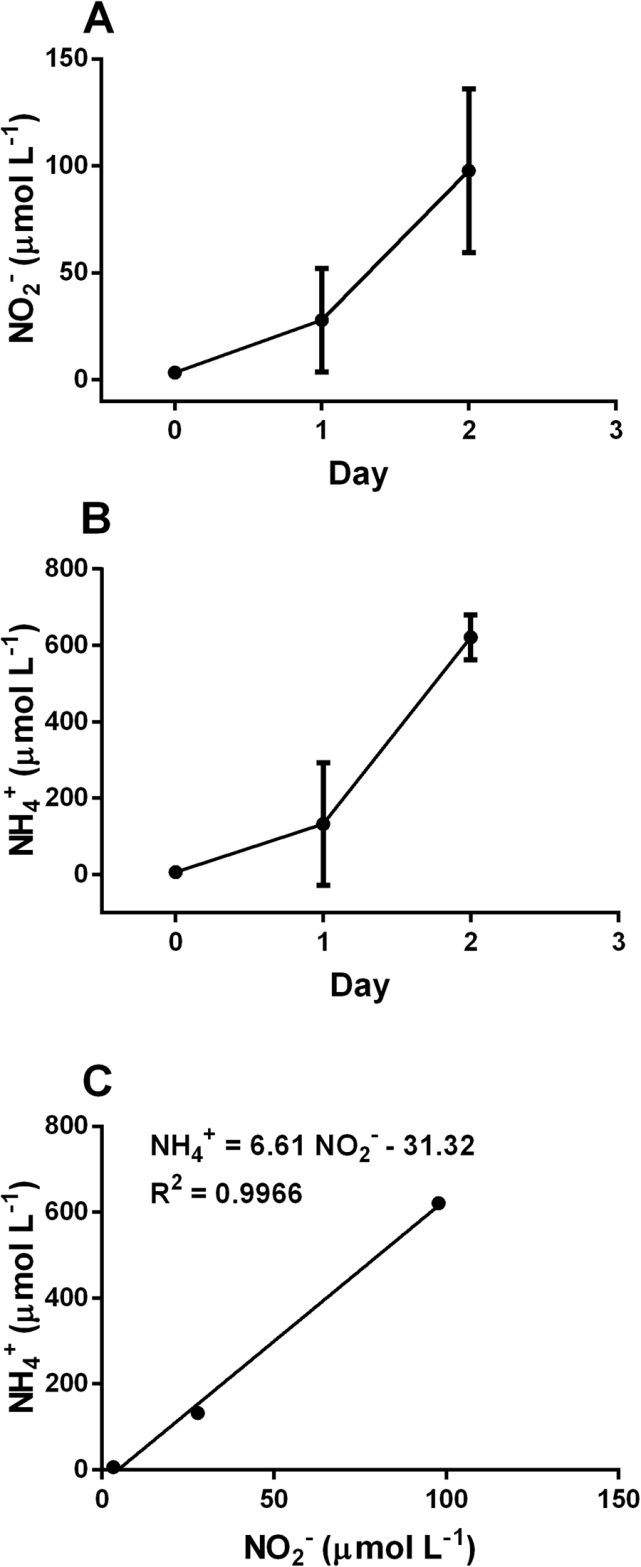
NO_2_^−^ (**A**) and NH_4_^+^ (**B**) concentrations (µmol L^−1^) along the exponential growth phase in the original f/2 medium with aeration; (**C**) correlation between NO_2_^−^ and NH_4_^+^ concentrations during the exponential growth phase.

Our study reveals that in the conventional f/2 medium used for diatom’s cultivation^41^ cells reach the stationary phase in parallel with the depletion of P and/or Si in agreement with previous studies^36,42^. PO_4_^3−^ concentration dropped down to <0.1 µmol L^−1^ (Fig. 1A,B) when *Skeletonema marinoi* reached a concentration of 500,000 cells mL^−1^. Also, SiO_4_^4−^ concentration strongly lowered, reaching <0.5 µmol L^−1^ (Fig. 1C), while NO_3_^−^ decreased along the growth curve, without becoming limiting (Fig. 1D). NO_3_^−^ concentration decreased in parallel with an increase of nitrite (NO_2_^−^) and ammonium (NH_4_^+^) concentrations (Fig. 2A,B), while the increases of NO_2_^−^ and NH_4_^+^ concentration were significantly correlated together (p < 0.01; Fig. 2C). The low decrease in NO_3_^−^ and the strong increase of NH_4_^+^ along the growth curve indicates that these elements are probably not the main N source of *S*. *marinoi*. It is surprising since NH_4_^+^ is considered as the preferred N source by many microalgae thanks its reduced state and energetically favourable assimilation^43^.

In culture medium, organic nitrogen coming from microalgal excretion occurring during death or lysis or also during productive growth phase can be a source for NH_4_^+^ production^44^. Urea is known as one of the main organic sources of NH_4_^+^ ^45^. Since the high biomass concentration and the elevated growth and division rate, we expect a high concentration of organic nitrogen (e.g. urea, amino acids) in the medium that in turn can be re-used by microalgae^46,47^. This hypothetic cartoon matches with the low decrease of NO_3_^−^ with time during high growth rate phase and agrees with results demonstrating that NH_4_^+^ in the medium has a negative effect on nitrate reductase enzyme (that catalyses the NO_3_^−^ reduction to NO_2_^−^) at both transcriptional and posttranscriptional levels in *Chlamydomonas reinhardtii*^20,48,49^.

These assumptions require further studies to better understand the biochemical cycle of N in diatom’s cultivation, since its relevance for the optimization of microalgal cultivation conditions.

These studies would focus on understanding the composition of the organic dissolved matter released in the medium, its evolution in time along the growth curve and on which nitrogen form diatoms do prefer and use when cultivating.

Nutrient uptake varied with growth rate (Table 2). Growth rate increase induced a decrease of NO_3_^−^ uptake (p < 0.05) and a PO_4_^3−^ uptake increase (p < 0.05). SiO_4_^4−^ uptake was almost stable. During the population death phase (negative growth rate class, Table 2), PO_4_^3−^ and SiO_4_^4−^ uptake significantly lowered (p < 0.01) conversely to NO_3_^−^ uptake, which remained stable compared to low positive growth rate class. The N:P ratio strongly decreased with the increase of growth rate (p < 0.01), while the N:Si ratio decreased slightly from low to high growth rate and the P:Si ratio little increased (Table 2). P concentration allocated to RNA was stable in the two positive growth rate classes (Table 2), while the total no-RNA-P content tended to increase, mainly due to the P-containing non-stored products (e.g., DNA, ATP, phospholipids).

**Table 2:** Nutrient uptake (pmol cells^−1^ d^−1^) and ratios between N:P, N:Si and P:Si uptaken by cells grown in f/2 medium with aeration.

Growth rate	N	P	Si	N:P	N:Si	P:Si	P-RNA	P-storage	P-no storage
−0.70 d^−1^ to 0.00 d^−1^ (n = 7)	0.37 ± 0.03	0.008 ± 0.001	0.01 ± 0.002	48.16 ± 4.80	25.46 ± 2.82	0.53 ± 0.07	0.017 ± 0.007	0.044 ± 0.02	0.176 ± 0.08
0.04 d^−1^ to 0.30 d^−1^ (n = 12)	0.39 ± 0.12	0.01 ± 0.006	0.1 ± 0.01	27.67 ± 4.98	3.91 ± 0.92	0.14 ± 0.05	0.044 ± 0.01	0.12 ± 0.02	0.48 ± 0.09
0.40 d^−1^ to 1.40 d^−1^ (n = 22)	0.19 ± 0.06	0.04 ± 0.02	0.07 ± 0.03	5.10 ± 1.86	2.82 ± 1.02	0.55 ± 0.23	0.038 ± 0.03	0.174 ± 0.14	0.696 ± 0.46

P-RNA (pg) is for P content allocation in RNA (using P = 0.091 g dry g RNA^−1^)^12^. P-storage (pg) is for P content allocation in reserve compounds (inorganic phosphate) and P-no storage (pg) is for P content allocation in other phosphorus-containing compounds. n is the number of data for each class of growth rate.

### On the role of water movement

**Figure 3:**
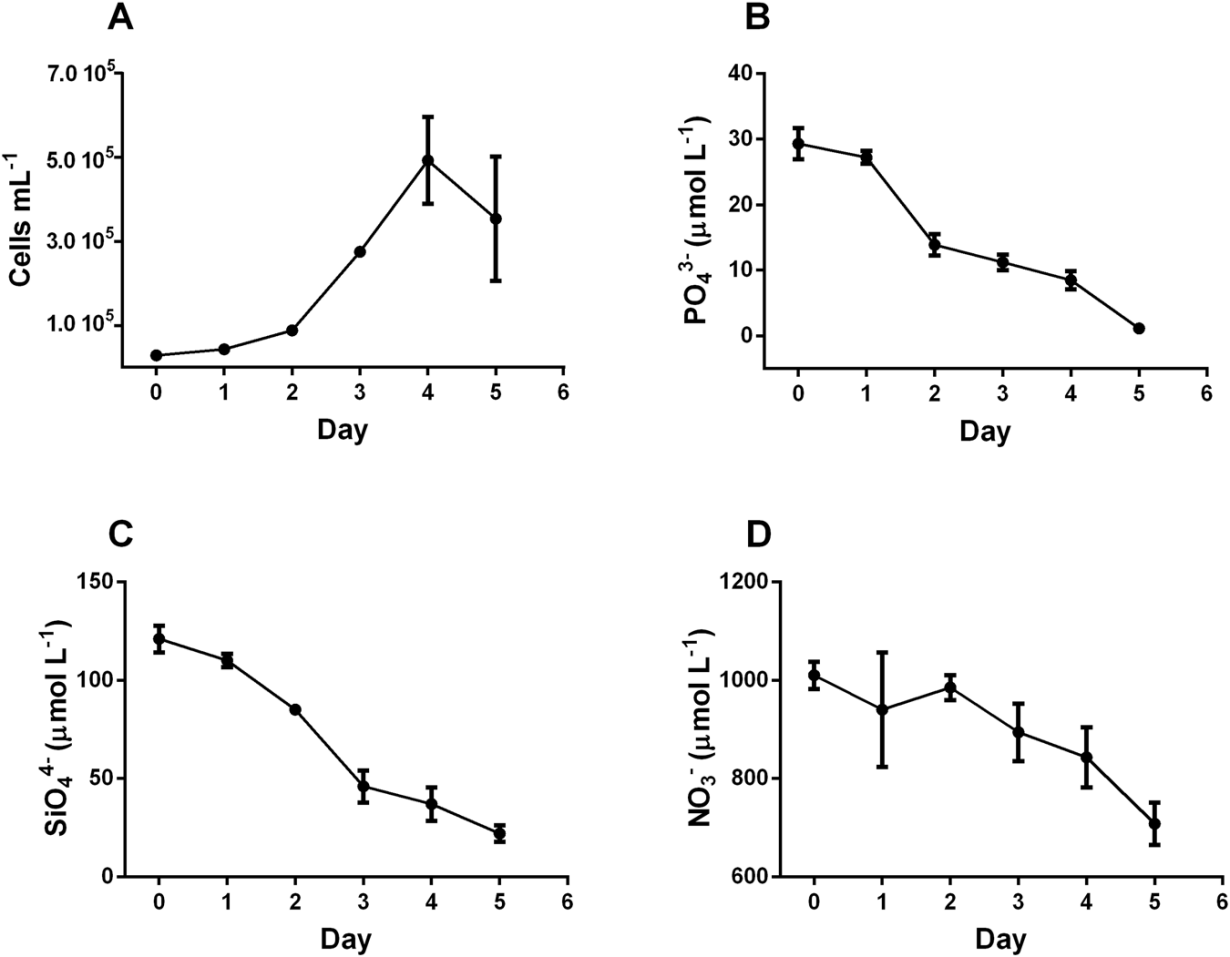
Cell concentration (**A)**, cells mL^−1^) and PO_4_^3−^ (**B**), SiO_4_^4−^ (**C**), and NO_3_^−^ (**D**) concentrations (µmol L^−1^) along the exponential growth phase in the original f/2 medium with water movement.

Using a wave marker pump instead of air-bubbling, growth rate was significantly higher (p < 0.01) compared to aeration (Fig. 3A), as reported by other studies^24,25,42,50^. While aeration ensures the exchanges between the medium and air thanks the addition of gas in the culture, water movement prevents sedimentation of the algae, ensures that cells are equally exposed to light^26^ and that they continuously explore new microscale environments^24,25^ with renewed nutrients composition/concentration, as well as improving gas exchange between the medium and the air. Dell’Aquila and co-authors^42^ hypothesized a better physiological state of cells grown under mixing compared to stagnant condition. Indeed, turbulence increases the chain length – used as proxy of a healthy physiological state^42^ - of the diatom *Chaetoceros decipiens*, and induces energy storage pathways like fatty acid biosynthesis^51^.

In parallel to growth rate enhancement, requirement in NO_3_^−^ and PO_4_^3−^ also increased. As in the previous culture condition, PO_4_^3−^ became limiting with concentration dropping down to <1 µmol L^−1^ (Fig. 3B), while SiO_4_^4−^ concentration decreased (Fig. 3C) but did not reach low values as observed during cultivation with aeration (Fig. 1C). NO_3_^−^ concentration remained high and was not limiting for growth (Fig. 3D).

Regrouping the data set in three growth rate classes (Table 3), it is noteworthy that SiO_4_^4−^ uptake significantly decreased (p < 0.01) with increasing growth rate, while PO_4_^3−^ uptake slightly increased. NO_3_^−^ uptake remained almost stable. The N:P ratio was significantly higher in the two positive growth rate classes compared to dying population class (p < 0.05), while it decreased with increasing growth rate (p < 0.05). N:Si ratio increased in parallel to growth rate (p < 0.05, Table 3), on the contrary to the previous observations reported in culture with aeration (Table 2). The P:Si ratio strongly increased with growth rate (p < 0.05, Table 3) as already found in the previous condition (f/2 with aeration).

**Table 3:** Nutrient uptake (pmol cells^−1^ d^−1^) and ratios between N:P, N:Si and P:Si uptaken by cells grown in f/2 medium with water movement.

Growth rate	N	P	Si	N:P	N:Si	P:Si
−2.45 d^−1^ to 0.00 d^−1^ (n = 6)	0.37 ± 0.02	0.09 ± 0.01	0.30 ± 0.06	4.18 ± 0.47	1.23 ± 0.18	0.29 ± 0.05
0.07 d^−1^ to 0.50 d^−1^ (n = 3)	0.75 ± 0.19	0.04 ± 0.003	0.48 ± 0.05	17.6 ± 2.96	1.54 ± 0.28	0.09 ± 0.01
0.60 d^−1^ to 1.20 d^−1^ (n = 13)	0.61 ± 0.04	0.06 ± 0.03	0.16 ± 0.08	8.95 ± 2.88	3.64 ± 1.04	0.40 ± 0.21

n is the number of data for each class of growth rate.

Comparing aeration and water movement, the trend of PO_4_^3−^ uptake with increasing growth rate is similar. Conversely, in efficiently growing cells, water movement induces a lowering of SiO_4_^4−^ uptake, as reported in another diatom^52^ and an increase of NO_3_^−^ uptake conversely of what it has been observed in the high growth rate class of cells submitted to aeration. Although the role of turbulence on microalgal nutrient acquisition has been the object of different studies^23–25^, few data on the three main nutrients together are available. More commonly, nutrient acquisition increases^46,50,52^ even though depending on algal species^25^, turbulence strength^24,25^ and the type of nutrients^52^. Our study also shows that differences in nutrient acquisition between different kinds of fluid circulation are strongly influenced by growth rate. Indeed, the significant link of NO_3_^−^ or PO_4_^3−^ acquisition to growth rate has been reported in some studies^30^ (also demonstrating the little influence of light condition compared to growth rate) by contrast to SiO_4_^4−^ acquisition^30,53^. Therefore, SiO_4_^4−^ uptake and its cellular use respond to other external or internal signals that the two other elements. As discussed in Spitzer^53^, Si requirement and metabolism in diatoms is variable and depends on species and cell size. Moreover, Levitan *et al*.^54^ report that Si-starvation is not physiologically growth damaging as N starvation. Another study^53^ reports that the silica present in frustules of two diatoms species is modulated with respect to the Si concentration in the media, without any variations of growth rate between high or low Si content. This suggests that cells efficiently growing preferably use internal Si pool for computing cell cycle progression and division, while Internal Si pool and its participation in the cellular metabolism respiration^11^ is controversial^9^.

### On the role of enriched medium together with water movement

**Figure 4:**
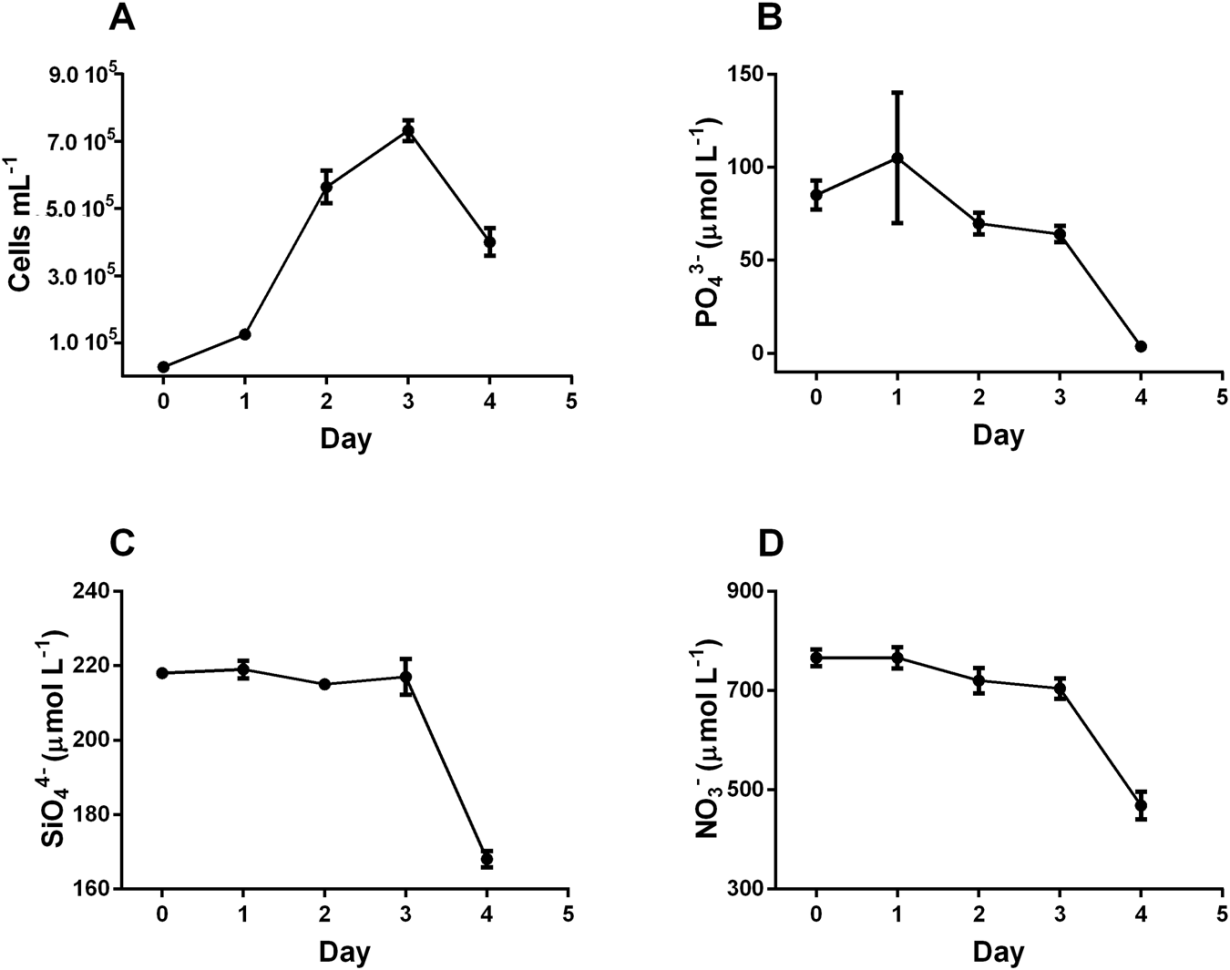
Cell concentration (**A**), cells mL^−1^) and PO_4_^3−^ (**B**), SiO_4_^4−^ (**C**), and NO_3_^−^ (**D**) concentrations (µmol L^−1^) along the exponential growth phase in the enriched medium with water movement.

Despite high cell concentration (≈ 750,000 cells mL^−1^; Fig. 4A), PO_4_^3−^ and SiO_4_^4−^ concentrations were consistently high, around 60 µmol L^−1^ (Fig. 4B) and ≈200 µmol L^−1^ (Fig. 4C), respectively. At the same cell concentration, NO_3_^−^ was still very high (700 µmol L^−1^, Fig. 4D) in the range of the concentration reported in the f/2 medium (Figs 1D, 3D). Enriching the medium prevents limitation of growth by nutrient depletion and culture reached the stationary phase independently of macronutrients’ limitation. Therefore, the population death phase is not explained, and different hypotheses might be advanced: from high level of intracellular reactive oxygen species produced during cell senescence, to external causes such as virus attacks or a death induced by a high concentration of toxic molecules (extracellular self DNA or sugars) in the medium such as observed in recent studies on higher plants or yeasts^55,56^. This last hypothesis well fit with the very high growth rate reached by cells, inducing a high excretion rate of products, and requires further studies.

The enriched medium induces a further and significant enhancement of growth rate (p < 0.01) compared to the original f/2 medium. Assumption is that the probability that cells encounter soluble nutrients to acquire is strongly increasing by doubling their concentrations together with water movement. The most relevant changes in nutrient acquisition in function of growth rate are noteworthy for PO_4_^3−^ and SiO_4_^4−^ (Table 4). Conversely, similar NO_3_^−^ uptake between enriched and conventional medium indicates that significant growth rate variations do not modulate NO_3_^−^ uptake suggesting and reinforcing the previous discussion on nitrogen acquisition under other N forms (urea, amino acids)^46^. Indeed, similar cellular NO_3_^−^ uptake is reported in the different growth rate classes (Table 4). PO_4_^3−^ and SiO_4_^4−^ uptake strongly decreased from low to moderate-high growth rate classes (p < 0.01, Table 4). As consequence, N:P and N:Si ratios increased from low to moderate/high growth rate (p < 0.05, Table 4).

**Table 4:** Nutrient uptake (pmol cells^−1^ d^−1^) and ratios between N:P, N:Si and P:Si uptaken by cells grown in an enriched medium with water movement.

Growth rate	N	P	Si	N:P	N:Si	P:Si	P-RNA	P-storage	P-no_storage
−4.70 d^−1^ to −0.01 d^−1^ (n = 42)	2.89 ± 0.40	0.67 ± 0.07	0.36 ± 0.05	4.29 ± 0.53	8.01 ± 1.11	1.87 ± 0.22	4.14 ± 0.32	3.34 ± 0.20	13.36 ± 1.00
0.00 d^−1^ to 0.70 d^−1^(n = 24)	0.71 ± 0.10	0.24 ± 0.03	0.25 ± 0.05	2.97 ± 0.39	2.77 ± 0.46	0.93 ± 0.14	0.15 ± 0.05	1.46 ± 0.41	5.84 ± 1.73
0.71 d^−1^ to 0.90 d^−1^ (n = 32)	0.59 ± 0.08	0.05 ± 0.004	0.09 ± 0.01	11.9 ± 1.32	6.03 ± 0.65	0.50 ± 0.04	0.09 ± 0.01	0.28 ± 0.03	1.12 ± 0.12
0.91 d^−1^ to 1.20 d^−1^ (n = 26)	0.82 ± 0.10	0.07 ± 0.01	0.10 ± 0.01	10.65 ± 1.85	7.56 ± 0.86	0.71 ± 0.11	0.07 ± 0.02	0.42 ± 0.10	1.68 ± 0.51
1.21 d^−1^ to 1.90 d^−1^ (n = 31)	0.72 ± 0.3	0.09 ± 0.02	0.04 ± 0.006	7.81 ± 1.50	16.7 ± 2.56	2.13 ± 0.26	0.04 ± 0.02	0.56 ± 0.30	2.24 ± 1.07

P-RNA (pg) is for P content allocation in RNA (using P = 0.091 g dry g RNA^−1^)^12^. P-storage (pg) is for P content allocation in reserve compounds (inorganic phosphate) and P-no storage (pg) is for P content allocation in other phosphorus-containing compounds. n is the number of data for each class of growth rate.

Comparing the three culture conditions, PO_4_^3−^ uptake is enhanced by water movement and further in enriched medium, such as growth rate, confirming the expected higher P requirement by cells with increasing growth rate, as also stated by the GRH. The three classes of P-containing compounds were significantly higher than in cells grown in the original f/2 medium (p < 0.05), although P allocation in cells varied with growth rate (Table 4). The P-RNA content surprisingly decreased with increasing growth rate (Table 4). Interestingly, in each growth rate class relationship between growth rate and P-RNA (phosphorus attributed to RNA) was significant (p < 0.001, Table 5) with the slope of the regression showing a decreasing trend from low to very high growth rate, except for the highest growth rate class (Table 5). This result is in agreement with the GRH^14,57^ and confirms growth rate as driver of the biochemical machinery of cells to run the genetic expression required by cells for cell cycle progression and division. The lack of robustness of the correlation between growth rate and P-RNA when pooling together all data from the different classes confirms that the complicated relationship between RNA content, P uptake and growth rate in photosynthetic organisms^13^. The intracellular P pool dynamics^13^ such as variations of P allocation into the different intracellular compounds (RNA, DNA, stored, etc.)^13^ along with growth phase might be the reasons for such complexity. For instance, in the low growth rate class – corresponding to cells in lag or post-exponential phase – the higher P uptake compared to the other classes might indicate an enhancement of the stored P pool or of the P used for repair activity.

**Table 5:** Enriched medium.

Growth rate	Correlation
−4.70 d^−1^ to −0.01 d^−1^	y = −7.21x − 3.57 R² = 0.54 (p < 0.001)
0.00 d^−1^ to 0.70 d^−1^	y = 0.62x + 0.027 R² = 0.57 (p < 0.001)
0.71 d^−1^ to 0.90 d^−1^	y = 0.36x − 0.028 R² = 0.89 (p < 0.001)
0.91 d^−1^ to 1.20 d^−1^	y = 0.12x − 0.004 R² = 0.41 (p < 0.001)
1.21 d^−1^ to 1.90 d^−1^	y = 0.29x − 0.26 R² = 0.79 (p < 0.001)

Spearman correlation between P-RNA (y, pg) and growth rate (x, d^−1^) for each of the growth rate class.

## Conclusions

This study assessed the effects of physical motion (water movement *vs*. air bubbling) and nutrient concentrations on the growth rate by the coastal centric diatom *Skeletonema marinoi*. Water movement enhanced growth rate and modulated nutrient acquisition rate. Enriched medium, compared to conventional f/2 medium, further enhanced growth rate and biomass harvesting. Results of this study contribute to enhancing diatom’s biomass productivity, reducing its cost, as it is required in the blue biotechnology context.
